# Efficacy and safety of low-frequency repetitive transcranial magnetic stimulation for bipolar depression: a study protocol for a multicenter, double-blind, randomized, sham-controlled trial

**DOI:** 10.3389/fpsyt.2025.1393605

**Published:** 2025-04-02

**Authors:** Takamasa Noda, Masako Nishikawa, Yuki Matsuda, Daisuke Hayashi, Shinsuke Kito

**Affiliations:** ^1^ Department of Psychiatry, National Center Hospital of Neurology and Psychiatry, Tokyo, Japan; ^2^ Clinical Research Support Center, The Jikei University School of Medicine, Tokyo, Japan; ^3^ Department of Psychiatry, The Jikei University School of Medicine, Tokyo, Japan

**Keywords:** bipolar depression (BD), rTMS (repetitive transcranial magnetic stimulation), low-frequency stimulation to the right prefrontal cortex, double-blind randomized sham stimulation-controlled trial, study protocol

## Abstract

**Introduction:**

Bipolar disorder has a long depressive episode and high risk of suicide. In clinical practice, patients often show no response to pharmacotherapy, which results in prolongation of the depressive episode. Repetitive transcranial magnetic stimulation (rTMS) is a non-invasive technique expected to serve as a treatment option for bipolar depression. For bipolar depression, a meta-analysis suggested that low-frequency stimulation to the right prefrontal cortex was possibly effective. However, a medium or large sample, randomized, double blind, sham controlled study has not yet been performed.

**Objective:**

To examine the efficacy and safety of 1-Hz rTMS to the right prefrontal cortex in patients with treatment-resistant bipolar depression. rTMS was approved by the Ministry of Health, Labor, and Welfare as a highly advanced medical technology on March 1, 2019.

**Methods:**

In this multicenter, double-blind, randomized, sham stimulation-controlled trial for bipolar depression, patients will be individually allocated to active or sham stimulation plus usual medication and followed up for 6 months. The conditions of stimulation by the Mag Pro R30 transcranial magnetic stimulation device (Magventure) will be a frequency of 1-Hz, intensity of 120% motor threshold, and duration of 1800 seconds to the right prefrontal cortex 5 days a week for 4 weeks during the acute treatment period. The primary endpoint will be a total change in the Montgomery-Åsberg Depression Rating Scale score during the acute treatment period.

**Discussion:**

The outcomes of this study will inform clinical practice for the treatment of bipolar depression.

**Clinical trial registration:**

https://jrct.niph.go.jp/latest-detail/jRCTs032180138, identifier jRCTs032180138.

## Introduction

Bipolar depression is a mood disorder consisting of depressive, manic, and hypomanic episodes. It has a high recurrence rate, rapidly advances to more severe episodes, severe disability, and frequently takes a chronic course (1, 2). Patients with bipolar depression also have a high risk of suicide attempt and a manic switch (3). Patients spend most of the time in a depressive state. According to the treatment guidelines developed by the Japanese Society of Mood Disorders, depressive episodes of bipolar depression (4) are treated with mood stabilizers or atypical antipsychotics, specifically quetiapine (300 mg/day), lithium (0.8 mEq/L of blood concentration), olanzapine (5–20 mg/day), lurasidone (20–60 mg/day), or lamotrigine (200 mg/day). However, in clinical practice, patients often show no response to pharmacotherapy, which results in prolongation of the depressive episode and difficulty in achieving remission. Other recommended treatments by the Japanese Society of Mood Disorders include the combined use of lithium (0.6–0.8 mEq/L of blood concentration) and lamotrigine (200 mg/day), therapeutic dose of lithium or valproic acid and lamotrigine, or electroconvulsive therapy (4). ECT requires pretreatment with intravenous anesthetics and muscle relaxants and may cause temporary memory impairment, including amnesia. Thus, it is necessary to develop therapeutic methods to treat intractable bipolar disorder given the limited number of treatment options. Transcranial magnetic stimulation (TMS) is a non-invasive technique that stimulates the brain through the generation of an eddy current, which appears in association with a variable magnetic field forming around a coil supplied with an electrical current. Delivery of the TMS pulses in short intervals is referred to as repetitive TMS (rTMS). High-frequency stimulation affects cortical excitability in a facilitatory manner (5), whereas low-frequency stimulation suppresses cortical excitability (6). In Japan, rTMS has been approved by the Ministry of Health, Labor, and Welfare as an insurance-covered therapeutic method for the treatment of refractory major depressive disorder. Many clinical studies used rTMS to treat refractory depression, but only a few studied bipolar disorders. To address this issue, Zengin et al. (7) showed that rTMS treatment to the left prefrontal cortex is effective and safe for 29 participants with treatment-resistant bipolar depression by randomized controlled trial. McGirr et al. (8) showed a double-blind, 4-week, randomized clinical trial for 37 participants of intermittent theta burst stimulation (iTBS) to the left dorsolateral prefrontal cortex (DLPFC). The iTBS to the left DLPFC was not efficacious in the treatment of bipolar depression. A systematic meta-analysis of randomized double-blinded comparative studies on depression, bipolar disorder, or both was performed to assess the treatment efficacy for bipolar disorder. A total of 19 clinical studies that met the quality criteria were included, involving 181 patients with bipolar disorder (9). This meta-analysis showed that the response rate for patients receiving active stimulation was 44.3%, whereas it was 25.3% for sham stimulation, suggesting that the treatment was effective in those receiving active stimulation for bipolar depression (9). The therapeutic efficacy was compared based on the number needed to treat (NNT); the NNT in the active stimulation group was 6 (95% confidence interval [CI], 4–15) compared to the control sham stimulation group. The NNT for high-frequency stimulation to the left prefrontal cortex was 7 (95% CI, 4–112), whereas that for low-frequency stimulation to the right prefrontal cortex was 3 (95% CI, 2–6) (9), suggesting that low-frequency stimulation to the right prefrontal cortex was possibly more effective. A single-blind randomized sham-controlled trial for 54 patients with antidepressant-nonresponding bipolar depression was performed (10). They concluded that 1-Hz 110% motor threshold and 300 pulses rTMS to the right prefrontal was not effective for bipolar depression over sham control. This failure was possibly attributed to a stimulus parameter (300 pulses/session for 15 sessions, with a total of 4,500 stimulation). Kito et al. conducted a preliminary study in which low-frequency stimulation was applied to the right prefrontal cortex and reported that the treatment was effective (11), safer, and more tolerable than high-frequency stimulation, a standard method of stimulation, applied to the left prefrontal cortex (11). The addition of 1-Hz 120% motor threshold and duration of 1,800 s (1,800 pulses) to the right prefrontal cortex rTMS to the usual medication will provide superior improvement of depressive symptoms compared to medication as usual for bipolar depression. However, a medium or large sample randomized, double-blind, sham-controlled study in this parameter has not yet been conducted. Therefore, as a therapeutic option for bipolar depression, we designed a medium sample, double blind, randomized, sham-controlled trial of low frequency (1,800 pulses/session for 20 sessions, with a total of 36,000 stimulation) to the right prefrontal cortex. This study aims to examine the efficacy and safety of 1-Hz rTMS to the right prefrontal cortex of patients with treatment-resistant bipolar depression. rTMS for bipolar depression was approved by the Ministry of Health, Labor, and Welfare as a highly advanced medical technology on March 1, 2019.

## Materials and methods

### Trial design

**Figure 1:**
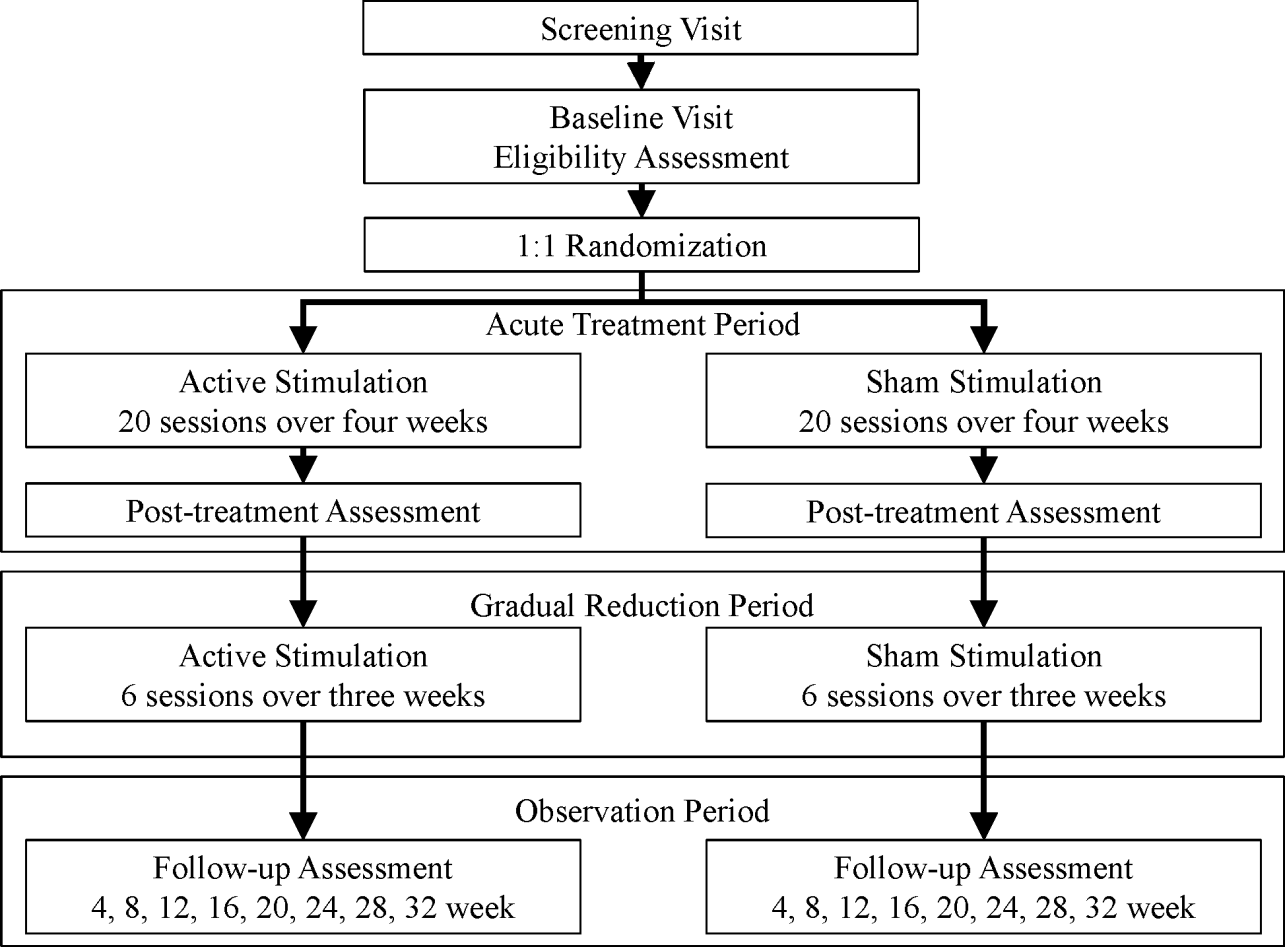
Flowchart summarizing the trial.

This study will be a multicenter, double-blind, randomized, sham stimulation-controlled trial. Patients will be individually allocated to 1-Hz rTMS plus usual medication (intervention group) or sham stimulation plus usual medication (control group) and followed up for 6 months. A summary of the EASyS-BD diagram is illustrated in **Figure 1**.

### Study setting

This study will comply with the Clinical Study Act (enacted in 2017, law number 16) and will be conducted with maximum attention paid to the dignity of individual study participants, respect of human rights, and protection of personal information. Participants will be recruited from three psychiatric departments: the National Center of Neurology and Psychiatry, Tokyo, Japan; Jikei University School of Medicine, Tokyo, Japan; and Keio University, Tokyo, Japan.

### Number of participants

Number of participants who obtained consent: active stimulation group, 48; sham stimulation group, 48; total, 96 (1:1 randomization).

Number of participants analyzed: active stimulation group, 38; sham stimulation group, 38; total, 76 (1:1 randomization).

A meta-analysis performed by McGirr et al. included patients with a depressive episode of bipolar disorder, and the effect size in the amount of change in depressive symptoms was 0.48 for low-frequency stimulation to the right prefrontal cortex (9). Since there were no previous studies on concomitant pharmacotherapy in bipolar disorder, the effect size was estimated as 0.65 based on the results of the aforementioned meta-analysis. The sample size was estimated assuming the effect size of 0.65, two-sided alpha equal 0.05, and power of 0.80. Overall, 80% of participants will be considered appropriate to be included in the double-blind study according to the inclusion and exclusion criteria. Therefore, with the sample size multiplied by 1/(1–0.20), the number of participants who will need to provide consent is 48 for each group; thus, we will need to recruit 96 participants.

### Recruitment

Outpatients from three medical institutions will be recruited. Advertising will be conducted through a web homepage and medical magazines to enhance recruitment.

### Eligibility criteria

Participants will be assessed for eligibility using the study-specific inclusion/exclusion criteria during the screening visit.

### Inclusion criteria

Patients will be included if they meet the following criteria:

1. Patients who meet the diagnostic criteria of bipolar disorder including types I and II with depressive episodes according to the *Diagnostic and Statistical Manual of Mental Disorders 5th Edition* (12) (DSM-5). However, in the case of a rapid cycler or mixed episode, the patient will be excluded from the study.
2. Patients aged 20 years or older and 75 years and younger at the time of consent.
3. Patients with a total 17-item Hamilton’s Rating Scale for Depression (HAMD-17) (13) score ≥18.
4. Patients who have had a depressive episode within the last 3 years.
5. During the current depressive episode, patients who have not responded to any of the following pharmacotherapies for more than 8 weeks without antidepressants (4).

 - Lithium, ≥0.8 mEq/L blood concentration
 - Quetiapine, 300 mg/day
 - Olanzapine, 5–20 mg/day
 - Lurasidone, 20–60 mg/day
 - Lamotrigine, 200 mg/day

The reason for setting each inclusion criterion is discussed below.

The reason for the use of HAMD-17 as an inclusion criterion is that a previous study conducted by O’reardon et al. (14). and other studies on magnetic stimulation employed HAMD-17 as an inclusion criterion and evaluated the efficacy with MADRS as the primary endpoint. The pilot study of refractory bipolar disorder conducted by Kito et al. also used HAMD-17 as an inclusion criterion and MADRS for the main efficacy evaluation. Thus, the reason for using HAMD-17 as an inclusion criterion is to be consistent with the large exploratory study of rTMS (conducted by O’reardon et al.), thereby enabling comparison. The reason for using MADRS instead of HAMD-17 for efficacy evaluation is that HAMD-17 has more parameters to evaluate sleep and allocates higher scores to the conditions than MADRS does. Therefore, similar to these evaluations, this study has also been designed to use HAMD-17 as an inclusion criterion and MADRS as the primary endpoint. Furthermore, we selected a total score of 18 or more in HAMD-17 because this was used as an inclusion criterion in the study of O’reardon et al. and because this study examines moderate or more severe bipolar disorders.

### Exclusion criteria

Patients will be excluded if they meet the following criteria:

1. Patients with a history of mental illness such as obsessive–compulsive disorder, post-traumatic stress disorder, or eating disorder.
2. Patients who have received neuromodulation such as ECT, rTMS, vagus nerve stimulation, deep brain stimulation, or transcranial direct current stimulation.
3. Pregnant patients.
4. Patients with a history of convulsive disorders such as epilepsy or patients whose close relatives (first-degree family members) have convulsive disorders.
5. Patients with a history of neurological disorders or organic brain disorders.
6. Drug- or alcohol-dependent patients.
7. Patients with magnetic material such as pacemakers, cochlear implants, or intracranial clips.
8. Patients with physical disorders such as serious metabolic diseases or endocrine disorders.
9. Patients with significant suicidal ideation (HAMD-17 score for suicide, ≥3).
10. Patients who are receiving pharmacotherapy that modifies the threshold of seizure (e.g., neuroleptics and tricyclic antidepressant agents).
11. Patients considered inappropriate by the investigator/physician representing the study.

### Randomization, allocation concealment

Cases will be enrolled and assigned to each stimulation group by the staff in charge of assignment in a centralized and aggregated manner. The subjects will be assigned with stratified block randomization. The stratified factors are sex and age (20–50, 51–75 years). Magventure will prepare the assignment table (corresponding table) on which six-digit patient codes are filled in, and the staff in charge of assignment will assign subjects to the actual or sham stimulation groups based on the assignment table. The staff in charge of assignment and the staff supporting the personal information management will not be allowed to recruit subjects, obtain IC, or perform intervention or neuropsychological testing.

The specific procedure is as below:

1. The staff in charge of recruiting study subjects and obtaining their informed consent (IC) will perform necessary assessment on the candidate patients according to the inclusion and exclusion criteria, after obtaining their written consent.
2. The case enrollment form on which prescribed information are filled in will be sent to the staff in charge of assignment to apply for enrollment and assignment.
3. After confirming the condition of the study subjects, the staff in charge of assignment will fill in the case enrollment number and six-digit patient code on the enrollment confirmation based on the assignment table prepared in advance and send it back to the study staff in each medical institution.
4. The study staff in each medical institution will receive the enrollment confirmation, which contains the case enrollment number and patient code, from the staff in charge of assignment.

### Blinding and unblinding

Patients, investigators, and the evaluator will be blinded. The emergency key for unblinding will be kept by the staff in charge, and assignment will not be disclosed, except for in emergency cases. Unblinding of the data will be performed only after completion of this study and fixation of the data. The emergency key for unblinding will be kept by the staff in charge of assignment and not be disclosed, except for emergency cases. Unblinding will not be allowed to determine future therapy.

### Intervention

#### Information on the medical devices

**Figure 2:**
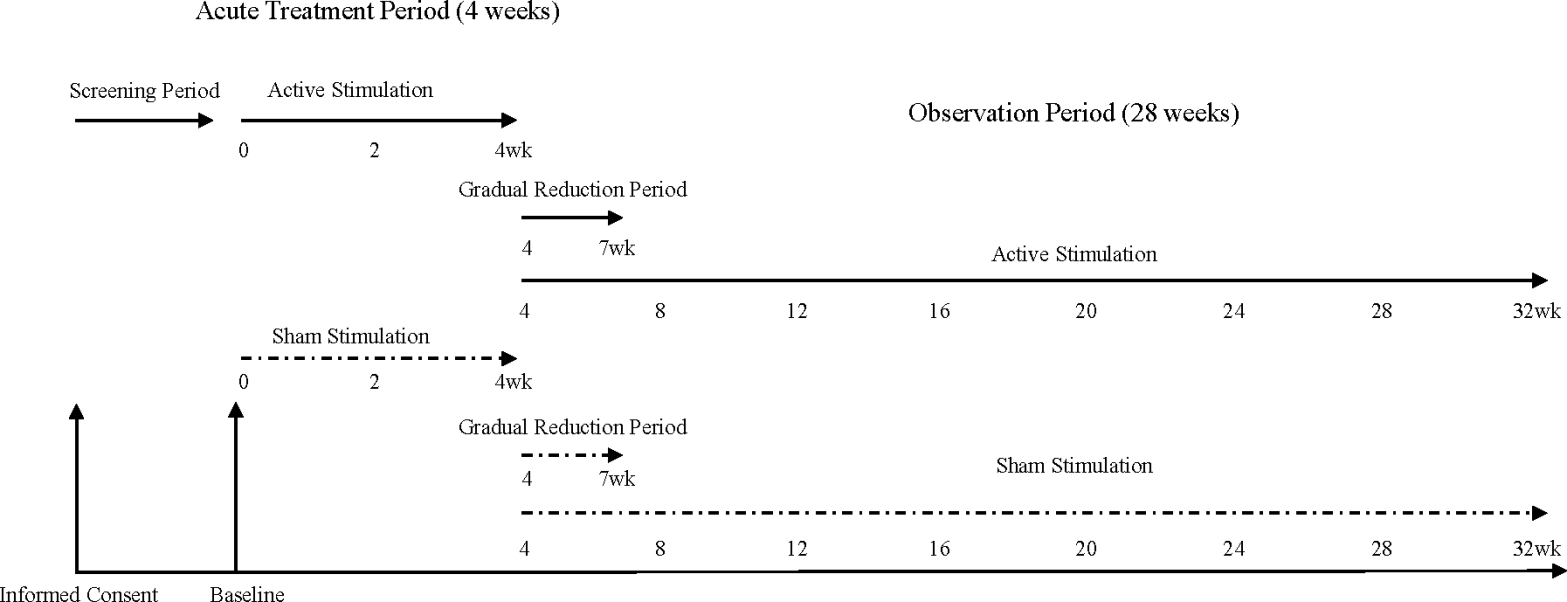
Schedule of informed consent, baseline, acute treatment period, gradual reduction period, observation period.

Based on a preceding study by McDonald (15), the conditions of stimulation by the Mag Pro R30 TMS device (Magventure) will be a frequency of 1 Hz, intensity of 120% motor threshold, and duration of 1,800 s (1,800 pulses) to the right prefrontal cortex, defined by the Beam F3 Location System (16), 5 days a week for 4 weeks during the acute treatment period, followed by a 3-week gradual reduction period (**Figure 2**). rTMS in a 3-week gradual reduction period will gradually reduce to 3 days a week in the first week, 2 days a week in the second week, and 1 day a week in the third week. Beam et al. have developed to find the F3 position using three skull measurement systems. The measurement of F4 (right DLPFC) is defined by Beam et al. (16) as follows:

1. Measurement of the distance from tragus to tragus and marking of the midpoint
2. Measurement of the distance from nasion to inion (the midpoint is marked here as well, and the vertex is the point where the two lines containing the midpoints meet)
3. Measurement of head circumference: Once these three measurements are attained, they are entered into the software package which provides two output values (values X and Y).
4. A point along the circumference is marked X cm from the midline.
5. F3 is marked as a point along the line running from the vertex through the point created in the previous step Y cm from the vertex.
6. The “x” at the top of the head is the vertex; the other “x” is the F3 location.
7. The opposite side of F3 from the midline is F4.

The stimulation coil Cool-B65 A/P (Magventure) will be used. This coil was developed for use in clinical studies, and its internal system allows for assignment to the active or sham stimulation group under double-blind conditions. Assignment to the active or sham stimulation will be determined by the six-digit patient code that will be assigned to each patient in advance. In the active and sham stimulation groups, a stimulating electrode will be placed on the participant’s scalp to deliver weak sensory stimulation in synchronization with pulses; thus, the participants will not be able to differentiate between sham or active stimulation. In sham stimulation, although the same sensory stimulation will be administered as in actual stimulation, the intensity will not be enough to reach the brain. This system will ensure a blinded process for participants receiving sham stimulation in the control group. Both participants and staff will be unable to determine whether the stimulation is active or sham.

### Conditions of concomitant drug and combination therapy

Medication such as lithium (>0.8 mEq/L blood concentrations), quetiapine (>300 mg/day), olanzapine (5–20 mg/day), lamotrigine (>200 mg/day), or lurasidone (20–60 mg/day), which will have been administered before the start of the study, and psychiatric therapy may be continued throughout the study under the same conditions. Additionally, eszopiclone at the maximum dose of 3 mg or zolpidem at the maximum dose of 10 mg as hypnotics and lorazepam at the maximum dose of 3 mg as anxiolytics will be administered as needed. ECT will be prohibited during the study period since it has a profound effect on the evaluation of the treatment efficacy.

### Evaluation

**Figure 3:**
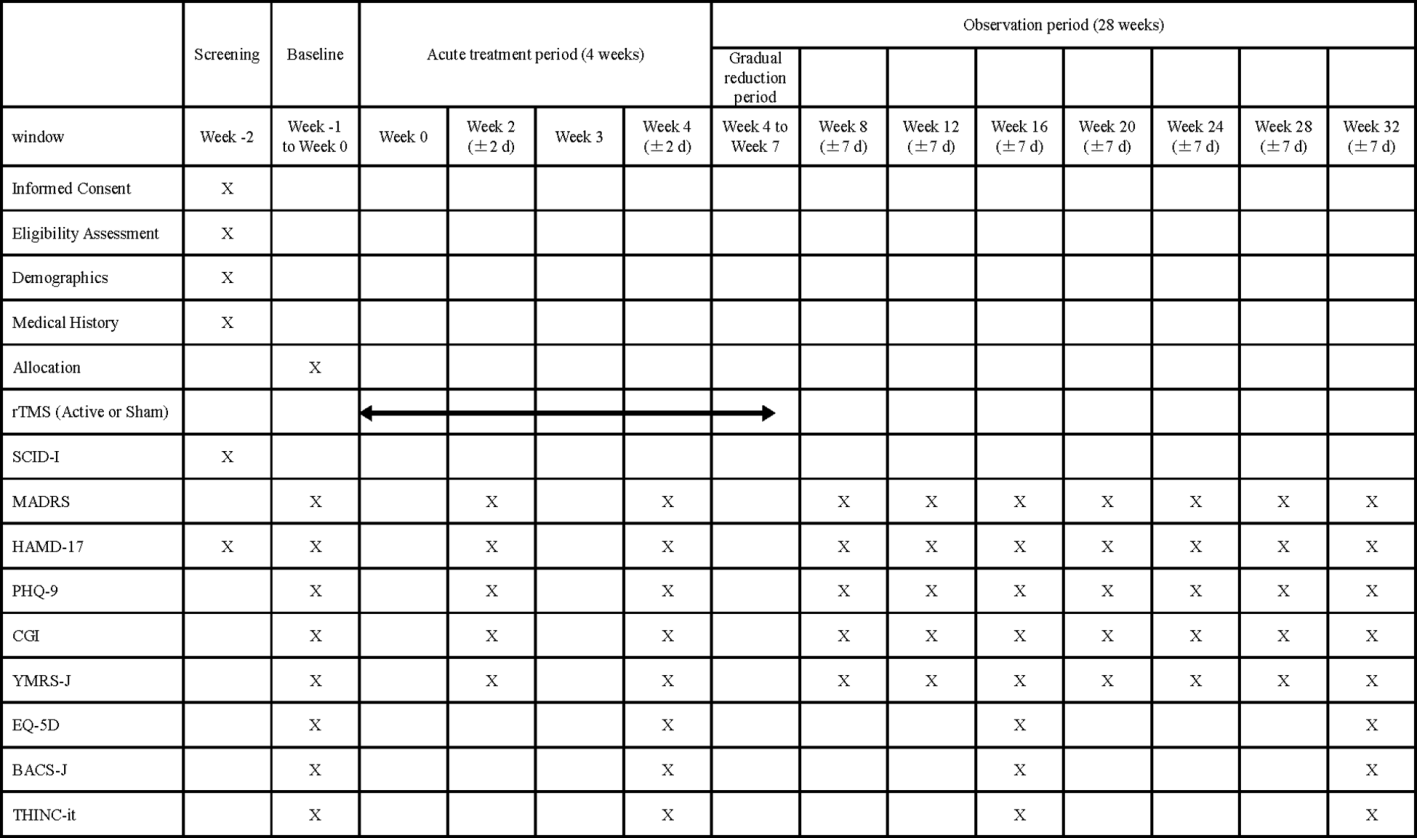
Standard protocol items: Schedule of enrollment, interventions, and assessments. rTMS, repetitive Transcranial Magnetic Stimulation; SCID-1, Structured Clinical Interview for DSM-4-TR Axis I Disorder; MADRS, Montgomery-Asberg Depression Rating Scale; HAMD-17, Hamilton's Rating Scale for Depression 17-item; PHQ-9, Patient Health Questionnaire; CGI, Clinical Global Impression; YMRS-J, Japanese version of Young Mania Rating Scale; EQ-5D, EuroQol 5 Dimensions; BACS-J, Brief Assessment of Cognition in Schizophrenia Japanese version; THINC-it, THINC-integrated tool.

The screening visit will commence after each participant has been enrolled. The schedule of examinations is listed in **Figure 3**.

1. Demographic variables and clinical observation information: Information including sex, age, main diagnosis, and disease duration are obtained from interview or medical records.
2. Psychological test parameters: face-to-face evaluation (interview)
3. Psychological test parameters: questionnaire
4. Psychological test parameters: cognitive functioning test
5. Assessment will be performed for items related to suicide in HAMD-17 and Young Mania Rating Scale (YMRS) for each rTMS delivery.

### Data quality

The same participant will be assessed by the same site evaluator throughout the study. A trained and qualified site rater will perform the efficacy and safety assessment. The inter-rater reliability (IRR) of the Montgomery–Åsberg Depression Rating Scale (MADRS) (17) will be confirmed with kappa coefficient. To improve IRR, all raters will be with a training trained using DVD and confirmed.

### Evaluation of adverse events

Safety parameters are evaluated for each rTMS stimulation. Information relating to the adverse event, date of onset, date of resolution, severity, treatment, outcome, seriousness, and relationship to rTMS will be described in the medical record and case report form. When the relationship to rTMS cannot be ruled out, follow-up observations will be performed as much as possible until recovery to normal conditions. The terms used to describe adverse events conform to those provided by the Medical Dictionary for Regulatory Activities/Japanese version (MedDRA/J): International Conference on Harmonization (ICH).

The safety evaluation parameters are as follows:

- Adverse events, particularly headache, stimulation site pain, stimulation site discomfort, presence or absence of muscle contraction (evaluated each time rTMS is delivered).
- The presence or absence of suicidal ideation (a parameter related to suicide in HAMD-17) and YMRS score (evaluated each time during the acute treatment and observation periods).

The results of the safety evaluation parameters should be recorded on the case report each time they are evaluated.

The severity of each adverse event is defined as follows:

- Mild: the condition in which the participant feels discomfort but does not interfere with daily life.
- Moderate: the condition that restricts the participant’s daily life and makes the participant feel uncomfortable.
- Severe: the condition wherein the participant is unable to work or perform daily activities.

### Compensation

If serious health hazards, including death, occur as a result of participation in this study, the participants can receive payment of compensation from the insurance that the researcher has purchased.

### Definition of terms

The terms of the endpoints of this study are defined as follows:

- Response: A response will be defined as a decrease of ≥50% in the HAMD-17 score measured at week 0 during the acute treatment period or a total HAMD-17 score of ≤12.
- Remission: The presence of remission will be defined as a total HAMD-17 score of ≤7.
- Relapse/recurrence: When a participant experiences conditions that meet the diagnostic criteria for depressive, manic, or hypomanic episodes defined in the DSM-5 after remission, during the acute treatment period, such conditions will be defined as relapse/recurrence. Additionally, relapse will be defined as disease exacerbation within half a year of remission, and recurrence will be defined as disease exacerbation after more than half a year of remission.

### Explanation of the terms

1. Hamilton’s Rating Scale for Depression (HAM-D).

This is a depression rating scale that was developed by Hamilton (24) and is used for measuring the severity of depressive symptoms in patients diagnosed with depression. The scale consists of a total of 21 parameters regarding depressive symptoms such as depressed mood or diminished interest and was confirmed to have clinical appropriateness and credibility by Hamilton (25).

2. Montgomery–Åsberg Depression Rating Scale (MADRS).

Montgomery and Åsberg developed the Comprehensive Psychopathological Rating Scale (CPRS) in Sweden, and the MADRS is a subscale of the CPRS, from which 10 parameters were extracted to evaluate depressive symptoms. This study used “the clinical evaluation of depression with the Japanese version of MADRS according to the Structured Interview Guide for MADRS (SIGMA)” (17).

3. Clinical Evaluation of Manic Disorders by the Japanese version of the YMRS.

This is a rating scale developed by Young et al. (20) for the clinical evaluation of manic episodes of mood disorders. The scale consists of 11 parameters related to manic episodes such as elevated mood and quantitative/qualitative increase in activity and has been confirmed to have sufficient credibility and clinical appropriateness.

4. Clinical Global Impression (CGI) (19).

This is a seven-stage clinical rating scale to evaluate the overall clinical impression.

5. Brief Assessment of Cognition in Schizophrenia, Japanese version (BACS-J).

This was developed by Keefe et al. (26) and comprises six tests of verbal memory, working memory, motor function, attention, verbal fluency, and executive function. The Japanese version was standardized in 2013 by Kaneda et al. (22).

6. THINC-it.

THINC-it is a screening tool developed to evaluate the cognitive function of patients with depressive episodes (23).

7. PHQ-9.

PHQ-9 is a self-completed rating scale developed to perform screening of depression, comprising nine parameters with four options. The Japanese version was examined for its translation, credibility, and appropriateness by Muramatsu (18).

8. EQ-5D (21).

EQ-5D is a self-completed rating scale developed to measure health-related QOL, comprising five parameters and three options.

### Case report form

All data will be entered into the Electronic Data Capture system (HOPE eACReSS). This system is compatible with the GCP requirements and allows for storage of the audit trail. The CRFs will not bear personal identifiable data including the participant’s name. Trial identification number will be used for identification. The study site will maintain a master participant identification log.

### Inspection of the source materials

Based on the descriptions in the protocol and separately prepared consent form, the physician representing this study, investigator, and head of medical institution where the study is conducted will be able to directly inspect all records related to this clinical study, including the source materials, at the time of monitoring and audit, as well as inspections conducted by the institutional review board and regulatory authority.

### Outcomes

#### Primary endpoint

The change in the MADRS total score from week 0 (starting point of rTMS) to week 4 will be used as the primary endpoint to clarify the treatment efficacy of rTMS in patients with bipolar disorder.

#### Secondary endpoints

The secondary endpoints will be as follows:

1. Response/remission maintenance rate during the observation period.
2. Relapse/recurrence rate during the observation period.
3. The time to relapse or recurrence during the observation period from the time origin is defined as end of the acute treatment.
4. The change in scores of the MADRS, HAMD-17, Patient Health Questionnaire-9 (PHQ-9) (18), Clinical Global Impression (CGI) (19), Young Mania Rating Scale (YMRS) (20), EuroQol 5 Dimensions (EQ-5D) (21), Brief Assessment of Cognition in Schizophrenia Japanese version (BACS-J) (22), and THINC-integrated tool (THINC-it) (23) during the acute treatment and observation periods.
5. Adverse events, particularly headache, pain at the stimulation site, discomfort at the stimulation site, and muscle contraction on stimulation.
6. Suicidal ideation (suicide subscale score of HAMD-17) and manic switch (YMRS score).

### Criteria for discontinuation of individual study participant

If the principal investigator, principal investigator, or sub-principal investigator determines that it is not feasible to continue research on an individual research subject for any of the following reasons, the research on that research subject will be terminated. With full consideration given to the ethical aspects of study subjects, information on observations and tests related to the primary and secondary endpoints will be collected after the point of discontinuation to the greatest extent possible.

1. Withdrawal of consent or request for withdrawal to participate by the study participants.
2. It is revealed after enrollment that the eligibility criteria were not met.
3. Suicidal ideation is exacerbated (HAMD-17 score for suicide, ≥3) and continuation of the study is considered unfavorable.
4. The total YMRS score is ≥8.
5. The occurrence of an adverse event makes it difficult to continue the study.
6. Pregnancy.
7. Significantly poor compliance (rTMS is performed 2 days or less in 2 consecutive weeks).
8. Occurrence of a convulsive seizure.
9. Relapse/recurrence during the observation period.
10. Discontinuation of the entire study, and
11. The investigator considers it appropriate to discontinue the study for other reasons.

### Statistical analysis

Statistical analysis will be conducted using the Statistical Analysis System software. All statistical tests will be two-sided, and the significance level will be set at 0.05. The primary analysis set of the efficacy and safety is the full analysis set (FAS), which will consist of all the randomized patients except those in whom neither active nor sham stimulation was performed. The secondary analysis set is the per protocol set (PPS), which will consist of all the eligible cases defined below.

### Definition of case classification

Eligible cases: the participants who meet all the inclusion criteria and do not meet any of the exclusion criteria.

Discontinued cases: the participants who discontinued the study according to the criteria of discontinuation of individual case.

Handling the data of discontinued cases or missing values:

- Discontinued cases
  For the FAS, the values measured until the discontinuation time will be used for evaluation irrespective of the discontinuation time. For the PPS, the discontinued cases will be treated as missing values if the participant discontinues the study <4 weeks from the start of the study. Measured values will be used if the participant discontinues the study >4 weeks after the start of the study.
- Missing values
  The primary endpoint, the MADRS total score, will be analyzed using mixed-effects models for repeated measures (MMRM) including those patients who had some missing values. Moreover, sensitivity analyses will be performed using single imputation methods such as baseline observation carried forward (BOCF) that performs an evaluation by substituting values measured at baseline for missing values and last observation carried forward (LOCF) by substituting the last measured value prior to the missing values. As for multiple imputation, distributions are assumed for the missing values with different parameters of the distribution according to conditions such as treatment methods. Additionally, a supplementary complete case analysis will be performed with participants having no missing data during the period of acute phase treatment.
  The time (days) to relapse or recurrence during the observation period, a secondary endpoint, will be defined as the interval (days) from the first day of the observation period to the date when the relapse or recurrence was identified. If a patient had not come to hospital before week 28 of the observation period, two types of handling of data will be considered; one is that the relapse or recurrence date will be regarded as 7 days after the final evaluation date, and the other is that the observation will be treated as censored on the final evaluation date. If no relapse or recurrence is confirmed at 28 weeks, the observation will be treated as censored on the day of 28 weeks.

### Analytical method

#### Primary endpoints

The change in MADRS total scores from weeks 0 to 4 during the acute treatment period will be analyzed using MMRM, in which the MADRS total score at week 0 as well as the interaction between the total score at week 0 and time point as continuous covariates (fixed effects), and the intervention, age, diagnosis, institution, time point, and interaction between the intervention and time point as categorical variables (fixed effects), and the response variables included will be the change in MADRS total scores measured at weeks 2 and 4. An unstructured covariance model will be fitted to intra-individual covariance structure, and Kenward–Roger degrees-of-freedom (DF) method will be applied to estimate the DF. The hypothesis test will be performed based on the least square estimates. Main comparison between the interventions will be performed by contrast at 4 weeks. As sensitivity analyses, analysis of covariance (ANCOVA) after LOFC, after BOCF for missing data at 4 weeks, and complete case ANCOVA will be performed. As secondary analyses, the change in the MADRS total score from weeks 0 to 4 will be analyzed using Wilcoxon rank-sum test. Interim analysis will not be performed.

#### Secondary endpoints

For patients in whom remission is noted at week 4 during the acute treatment period, the days to relapse/recurrence from week 0 during the observation period will be analyzed using the Kaplan–Meier method and log-rank test.

Cox proportional hazards regression analysis will be performed, in which the MADRS total score at week 0, intervention, diagnosis, age, and institution will be explanatory variables.

#### Other endpoints

The response and remission rates at week 4 in the acute treatment period and at week 28 in the subsequent observation period will be analyzed using logistic regression analysis, in which the MADRS total score at week 0, intervention, diagnosis, age, and institution will be explanatory variables. As a secondary analysis, Fisher test will be used. For MADRS, HAMD-17, PHQ-9, CGI, YMRS, EQ-5D, BACS, and THINC-it, a comparison between the active and sham stimulation groups will be performed using Wilcoxon rank-sum test at week 4 during the acute treatment period and at week 28 in the subsequent observation period, respectively.

### Adverse events

The number of cases and proportion of adverse events in the active and sham stimulation groups will be calculated during the acute treatment period and in the subsequent observation period, respectively. Both groups will be compared and analyzed using chi-square test or Fisher test.

### Monitoring and audit

#### Monitoring

An independent monitoring staff will perform data monitoring to check the progress of the study and check if the study is conducted in compliance with the latest protocol, ethical guidelines on medical research involving human participants, clinical study act, and procedures. Moreover, they will check if the study data reported by the researchers are accurate and verify the source documents of study-related records.

#### Audit

An audit will be conducted periodically by a designated external organization.

### Periodic report to the Clinical Research Review Board

The principal investigator (PI) will report the progress of the study once a year and receive evaluation by the National Center of Neurology and Psychiatry Clinical Research Review Board on whether continuation of the study is appropriate. In the case of diseases or deficits caused by this study, the PI will report them to the hospital director and the Clinical Research Review Board on whether continuation of the study is appropriate.

### Protocol amendments

A revised protocol will be discussed and approved within the National Center of Neurology and Psychiatry Clinical Research Review Board (CRB3200004) and Ministry of Health, Labour, and Welfare.

## Discussion

Bipolar depression takes a chronic course (1–3). The depressive episode has a long duration (1, 2), and patients often show no response to pharmacotherapy, which results in prolongation of the depressive episode and difficulty in achieving effective treatment. It is necessary to develop therapeutic options to the treatment-resistant bipolar depression. Kito et al. conducted a preliminary study in which low-frequency stimulation was applied to the right prefrontal cortex, and they reported that the treatment was effective (7). Therefore, we designed a medium-sample-size, double-blind, randomized, sham-controlled trial of low-frequency stimulation to the right prefrontal cortex. Low-frequency rTMS stimulation to the right prefrontal cortex is possibly more effective, safer, and tolerable than high-frequency stimulation applied to the left prefrontal cortex. The results of this study may provide a treatment option for future clinical practice to patients with bipolar depression.

## Conclusion

This study is a prospective, multicenter, double-blind, randomized sham-controlled trial for only bipolar disorder. The stimulation parameter is set at 1-Hz low frequency to the right prefrontal cortex for bipolar disorder. The primary endpoint is total change in MADRS score during the acute treatment period. The outcomes of this study will contribute to clinical practice for the treatment of bipolar depression.

## Trial status and dissemination

The current protocol version is 3.2, dated February 27, 2023. The start date was March 1, 2019, and this study is currently recruiting. Recruitment is expected to be completed by February 28, 2027. The results will be published by March 31, 2029.
